# New insights on the role of some biometals in mancozeb induced hematological and hepatorenal toxicity in rats and the protective effect of naringin

**DOI:** 10.1007/s10534-026-00795-w

**Published:** 2026-02-25

**Authors:** Sara S. Elbagwry, Eman I. Hassanen, Rehab A. Azouz, Marwa A. Ibrahim, Rawhia Doghaim

**Affiliations:** 1 Department of Pathology, Faculty of Veterinary Medicine, Egyptian Chinese University, Cairo, Egypt; 2 Department of Pathology, Faculty of Veterinary Medicine, Cairo University, P.O. Box 12211, Giza, Egypt; 3 Department of Toxicology and Forensic Medicine, Faculty of Veterinary Medicine, Cairo University, Giza, Egypt; 4 Department of Biochemistry, Faculty of Veterinary Medicine, Cairo University, Giza, 12211 Egypt

**Keywords:** Flavonoid, Fungicide, Gene expression, Immunohistochemistry, Oxidative stress, Essential elements

## Abstract

Prolonged exposure to mancozeb (MZ), a frequently used fungicide, may cause oxidative stress damage to several organs; however, the mechanism of toxicity remains obscure. So, the present work sought to assess the contribution of some biometals, including Mn, Zn, Ca, and Fe, to the subacute hepatic and renal injury prompted by MZ-mediated oxidative stress and to evaluate the protective impact of naringin (NAR), a citrus-derived flavonoid, against this toxicity. Twenty-eight male Wistar rats were divided into four groups (n = 7) as follows: (1) control, (2) NAR (20 mg/kg bwt), (3) MZ (250 mg/kg bwt), and (4) NAR + MZ. The daily oral intake of MZ for 54 days induced marked hematological alterations, elevation in some hepatorenal markers, and alteration of the redox status of both liver and kidney tissues. There were marked histopathological alterations in both liver and kidney tissues that were confirmed by the immunohistochemical staining, which demonstrated strong iNOS and Bax along with weak Bcl-2 immunoexpression. The repeated exposure to MZ significantly increased the levels of Mn and decreased the levels of Ca, Zn, and Fe in both liver and kidney tissues, which attributed to upregulation of the mRNA levels of *MT-1, CYP1A1*, and *casp-3* genes. On the other hand, the co-administration of NAR with MZ significantly reversed these toxic effects via improving the hematological profile, restoring antioxidant enzyme activity, and mitigating both hepatorenal function and structure. The data indicated that NAR had considerable protective effects against MZ-induced hepatorenal damage, mostly through enhanced antioxidant capacity, preservation of trace element equilibrium, and modulation of oxidative, inflammatory, and apoptotic pathways.

## Introduction

Mancozeb (MZ), an organometallic polymeric complex of manganese/zinc ethylene-bisdithiocarbamate (Mn/Zn-EBDC), considered as an often-used fungicide in agriculture to control a variety of fungal infections of ornamental plants and vegetables (Abdoon et al. 2011). MZ is regarded as a broad-spectrum fungicide widely used in homes, farms, and industries (Abdelkader et al. 2023). Distinct levels of MZ residues remained in various food commodities like fruits (1.33–1.63 mg/kg), vegetables (0.03–0.80 mg/kg), and their processed or dried products either pre- or post-harvest (Naman et al. 2022; Sharma et al. 2024).

Agricultural workers are the most at risk of MZ exposure after skin contact, inhalation of contaminated dusts, or accidental ingestion, like eating without prior handwashing (Runkle et al. 2017). Many recent studies have proved the significant toxic effects of MZ on various organs, mainly kidneys and liver (Gök and Deveci 2022; Akhtar and Trombetta 2023). Recent studies have proved that MZ induces significant oxidative stress in various non-target organisms across aquatic, earthlike, and plant systems (Kumar et al. 2023). In fish, MZ exposure disrupts redox balance, leading to elevated lipid peroxidation, suppressing antioxidant enzymes and developmental abnormalities (Vieira et al. 2020). MZ exposure impairs antioxidant defenses and induces neurotoxicity (Ansari and Ismail 2012). Oxidative stress is a central mechanism underlying MZ toxicity which is implicated in mitochondrial dysfunction, DNA damage, and systemic physiological disruption in non-target species (Silver et al. 2010).

Naringin (NAR), a flavanone-7-O-glycoside derived from citrus fruits particularly grapefruits, is responsible for the fruits' bitter taste (Chen et al. 2016). A comprehensive analysis of the literature has revealed that NAR owns several biological properties, such as antioxidants, anti-inflammatory, anti-ulcer, anti-osteoporotic, anti-apoptotic and anti-carcinogenic effects (Jiang et al. 2023; Peng et al. 2024). Several experimental studies have proven that NAR showed protective effects against oxidative stress by efficiently scavenging ROS, enhancing endogenous antioxidant defense mechanisms, and neutralizing transition metals in different biological systems (Khaled et al. 2023; Salama and El Shahed 2024). Ali et al. (2020) reported that NAR decreasing nephrotoxicity induced by iron overload in rats by reducing lipid peroxidation and chelating excess iron, suggesting effective role as an antioxidant and metal chelator. Another study highlighted the capacity of NAR to remove excessive iron in the hippocampus of iron-overloaded mice, which showed neuroprotective potential (Jahanshahi et al. 2021). Naringin's antioxidant effects were investigated against lambda-cyhalothrin in rats, while results say that NAR efficiently reduces oxidative stress damage and recovers antioxidant defense mechanisms that have been reduced by pesticide exposure (Xu et al. 2023). Moreover, Gerçek et al. (2021) investigated the antioxidant capacity of NAR against malathion-induced oxidative stress by scavenging activity and enhancing endogenous antioxidant defense systems.

Despite increasing research on MZ toxicity in experimental animals, the mechanism of toxicity is still not fully explained. Therefore, the current study aimed to explain the role of some biometals including manganese, zinc, iron, and calcium on MZ-induced hepatorenal toxicity in rats. It also investigated the role of Bax, Bcl-2, iNOS, *caspase-3, Metallothionein (MT-1), and Cytochrome P450 (CYP1A1)* in the underlying molecular mechanisms and explored the cytoprotective potential of NAR against such toxicity.

## Materials and methods

### Chemicals and kits

Commercial fungicide formulation of Mancozeb (85% WP, manganese ethylenebis, Di thiocarbamate, polymeric, complex with zinc salt, C8H12MnN4S8Zn) was obtained from Dow Agro sciences, France. It was dissolved in sesame oil according to the required concentration. Naringin (purity ≥ 98%, CAS 10236-47-2) was obtained from Santa Cruz biotechnology, USA. It was dissolved in methanol and then diluted with deionized distillated water according to the required dose. Aspartate aminotransferase (AST) kit (Cat. # AS 1061), Alanine aminotransferase (ALT) kit (Cat. # AS1031), creatinine (Cat. # CR 12 50) kit, blood urea nitrogen (BUN) kit (Cat. # UR 21 10), and total protein (TP) kit (Cat. # TP 20 20) were bought from Biodiagnostic, Cairo, Egypt.

### Animals and experimental design

Experimental techniques and protocols were carried out in accordance with the ARRIVE guidelines and the UK Guidance on the operation of the Animals (Scientific Procedures) Act 1986 as well as approved by the Institutional Animal Care and Use Committee (IACUC) of Cairo University (approval ID: Vet CU131020241057).

Twenty-eight adult male albino Wistar rats (150 ± 20 g) were bought from the Animal House of the VACSERA, Helwan, Egypt. The rats were housed in plastic cages and provided with standard commercial pelleted feed that obtained from Al-Watania Com. for Fodder, El-Dakahleya, Egypt. They had unrestricted access to water throughout the experimental period. The standard temperature (21–23 °C), humidity (40–60%), and a daily 12/12h light/dark cycle were all kept. Before receiving treatment, the animals underwent health evaluations and were allowed a two-week acclimation period to adjust to the laboratory environment.

Rats were randomly assigned to one of four groups (n = 7) and administered the indicated substances every day via gastric intubation for 54 days. Group 1 (control) was given sesame oil and served as the negative control. Group 2 (NAR) was given NAR at a dose of 20 mg/kg body weight. Group 3 (MZ) was given MZ at 250 mg/kg bwt, which is 1/20 of LD50. Group 4 (MZ + NAR) was treated with both MZ and NAR (one hour in between) at the same formerly mentioned doses. The pesticide dose was chosen according to their oral LD50 in rats, which was documented as 5000 mg/kg bwt (Yahia et al. 2019). While the dose of NAR was chosen based on an earlier study (Kumar et al. 2013). Each group was examined every day to record any abnormal symptoms, and both the initial and final body weights were also recorded.

### Sampling

Rats were anesthetized at the end of the experiment (54 days) with a single intramuscular injection of xylazine (10 mg/kg) and ketamine (90 mg/kg) prior to blood sample collection from the orbital sinus. A part of blood was collected in heparinized tubes for CBC, while the other part centrifuged at 3500 rpm for 5 min to acquire serum samples that were stored at − 21 °C. Following euthanasia by decapitation, hepatic and renal tissues were also collected. A portion of each sample was stored at − 80 °C for biomarkers assay, element residual analysis, and gene expression assay. While the remaining portion was preserved in 10% neutral buffered formalin for immunohistochemical and histopathological examination.

### Hematological profile

Some Hematological parameters including RBC count, hemoglobin levels, total and differential leukocytic count, and platelet count were performed on fresh blood samples using Giemsa stain for visual examination and Erma Hematology Analyzer (Tokyo, Japan) as previously described (Bain et al. 2016).

### Biochemical analysis

Serum concentrations of AST, ALT, creatinine, BUN, and total protein were determined using the guidelines provided with the manufacturer's kits (Biodiagnostic, Cairo, Egypt).

### Oxidative stress evaluation

Frozen tissue samples from the liver and kidney were homogenized in a cold potassium phosphate buffer (pH 7.4) holding 1 mM EDTA. The homogenates were then used to evaluate oxidative and antioxidant biomarkers, including malondialdehyde (MDA), catalase (CAT), and glutathione reductase (GR), following the protocols provided by the assay kits (Biodiagnostic Co., Egypt).

### Histopathological examination

Formalin-fixed liver and kidney tissue samples were processed using an ascending series of alcohol and xylene. The specimens were then embedded in paraffin, sectioned to 4.5 μm thickness with a standard microtome, stained with H&E, and examined under an Olympus BX43 light microscope. Images were captured using an Olympus DP27 camera connected to Cell-Sens Dimensions software and anonymized codes were used for all digital images (Bancroft and Layton et al. 2013).

To evaluate the distribution of the histological lesions across hepatic and renal sections of various experimental groups, a multiparametric semi-quantitative grading system was employed. Two independent pathologists, blinded to the experimental groups, assessed liver and kidney abnormalities (cellular degeneration, necrosis, and inflammatory cells infiltration). Five images per slide across seven slides/group (representing seven rats) were assessed using a scale of 0 (normal histology) to 4 (extensive severe damage, > 75% tissue damage), with intermediate scores of 1 (mild, < 25%), 2 (moderate, 25–50%), and 3 (severe, 50–75%). Finally, the total score index for each organ was calculated by summing its lesion scores (Hassanen et al. 2025).

### Bcl-2, Bax and iNOS immunostaining

Hepatic and renal sections were incubated with Bcl-2, Bax, and iNOS primary antibodies (Abcam, Ltd.) at a 1/200 dilution, followed by treatment with Peroxidase Block (Sakura BIO) and a reagent for detecting the antigen–antibody complex (Power-Stain 1.0 Poly HRP DAP Kit; Sakura). After incubation with 3′diaminobenzidine chromogen substrate for 10 min, the sections were counterstained with hematoxylin and examined under a light microscope (Olympus BX43) with images captured by an Olympus DP27 camera. Quantitative scoring for each immune marker was done through measuring the mean percentage of positively stained area in at least 15 images/group using Image J software.

### Elemental analysis

Concentrations of Mn, Fe, Ca, and Zn in both the hepatic and renal tissues were measured in accordance with the AOAC Official Method 985.01 using inductively coupled plasma optical emission spectrometry (ICP-OES). Fresh hepatorenal tissue samples were first accurately weighed, then digested using a mixture of concentrated nitric acid and perchloric acid under controlled heat until a clear solution was obtained. The digested samples were then diluted to a known volume with deionized water. Standard solutions of known metal concentrations were prepared to construct calibration curves. The concentrations of the elements in the samples were measured at their specific wavelengths using the ICP-OES instrument. Quality control procedures included the use of reagent blanks and certified reference materials to ensure accuracy and precision of the results (Peña-Vázquez et al 2007).

### Quantitative RT-PCR analysis of* MT-1*,* CYP1A1*, and* caspase-3* expression

The total RNA was extracted from liver and kidney samples using the RNeasy Mini Kit (Qiagen, Catalog No./ID: 74104). The synthesis of first-strand complementary DNA (cDNA) was performed using SuperScript Reverse Transcriptase (Thermo Scientific) following the manufacturer's protocol. Quantitative real-time PCR (qRT-PCR) was conducted using the SYBR Green PCR Master Mix (Thermo Scientific, Catalog No. 4309155) and an ABI Prism StepOnePlus Real-Time PCR System (Applied Biosystems), following the recommended instructions (El-Shiekh et al. 2024). Specific primer sets for the target genes are detailed in Table 1. The qRT-PCR protocol involved an initial denaturation step at 95 °C for 5 min, followed by 35 amplification cycles consisting of 30 s at 95 °C and 30 s at 58 °C. Melting curve analysis was performed by gradually increasing. the temperature from 65  to 90 °C to confirm the specificity of amplification products. Each sample was analyzed in duplicate to ensure reproducibility, and no-template controls were included to validate the assay's accuracy**.** Normalization of gene expression levels was achieved using the ACTB gene as an internal control. The relative quantification method employed was based on the approach outlined by Abdelrahman et al. (2025).

**Table 1:** Primer sequence of the studied genes

Primer	Sequence	Amplicon size (bp)	Accession number	Reference
*CYP1A1*	f-GCAAAAGGTCTTTGCCTGCG r-TGGATTCTGTGTGTGCCGTT	212	NM_017286.3	Moselhy et al. (2024)
*Caspase 3*	F:CATGCACATCCTCACTCGTG R: CCCACTCCCAGTCATTCCTT	158	NM_012922.2	Abdelrahman et al. (2024)
*MT-1*	f-TGTCGCTTACACCGTTGCTC R-CACTTGTCCGAGGCACCTTT	210	NM_138826.4 R	–
*ACTB*	F:CCGCGAGTACAACCTTCTTG R:CAGTTGGTGACAATGCCGTG	297	NM_031144.3	AbdElrazek et al. (2023)

*CYP1A1* Cytochrome P450-1A1, *MT-1* Metallothionein, *ACTB* beta-actin housekeeping gene

### Statistical analysis

Data are presented as mean ± standard error of mean (SEM) and were analyzed using SPSS version 27.0 (SPSS Inc., Chicago, IL, USA). One-way ANOVA and Tukey’s post hoc test was used to compare group means, with *P* ≤ *0.05* considered statistically significant. While the nonparametric data (histopathological scoring) are presented as median with range and analyzed using Kruskal Wallis-H test and Dunk’s test.

## Results

### Body weight gain (BWG), clinical signs, morbidity, and mortality

**Fig. 1:**
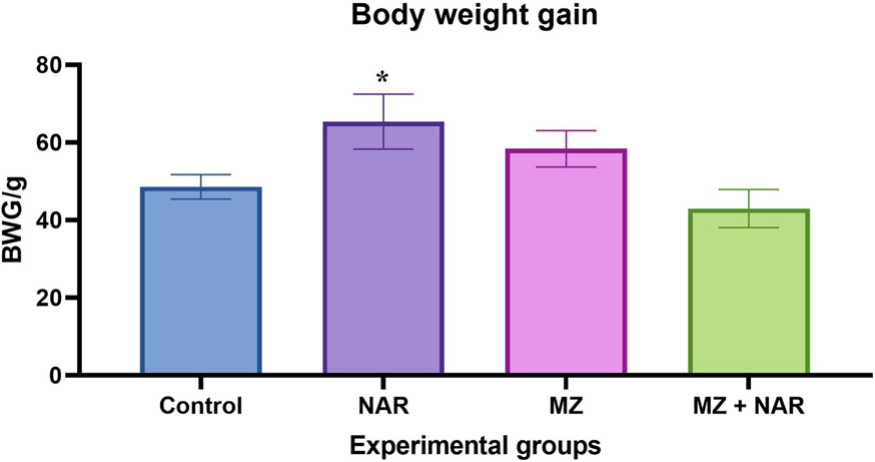
Bar chart represents the effect of MZ and/or NAR on the body weights gain (BWG) of rats. All data are presented as mean ± SE (n = 7 rats/ group). ⁎ means a significant difference between groups at *P* ≤ *0.05*

Throughout the experimental period, rats in the diverse groups did not show any distinct clinical symptoms or mortality. Concerning body weight, administration of NAR significantly increased the BWG in contrast to the other groups. Moreover, the MZ group didn’t show any significant difference in the BWG compared to either the control or NAR + MZ group (Fig. 1).

### Hematological parameters

**Fig. 2:**
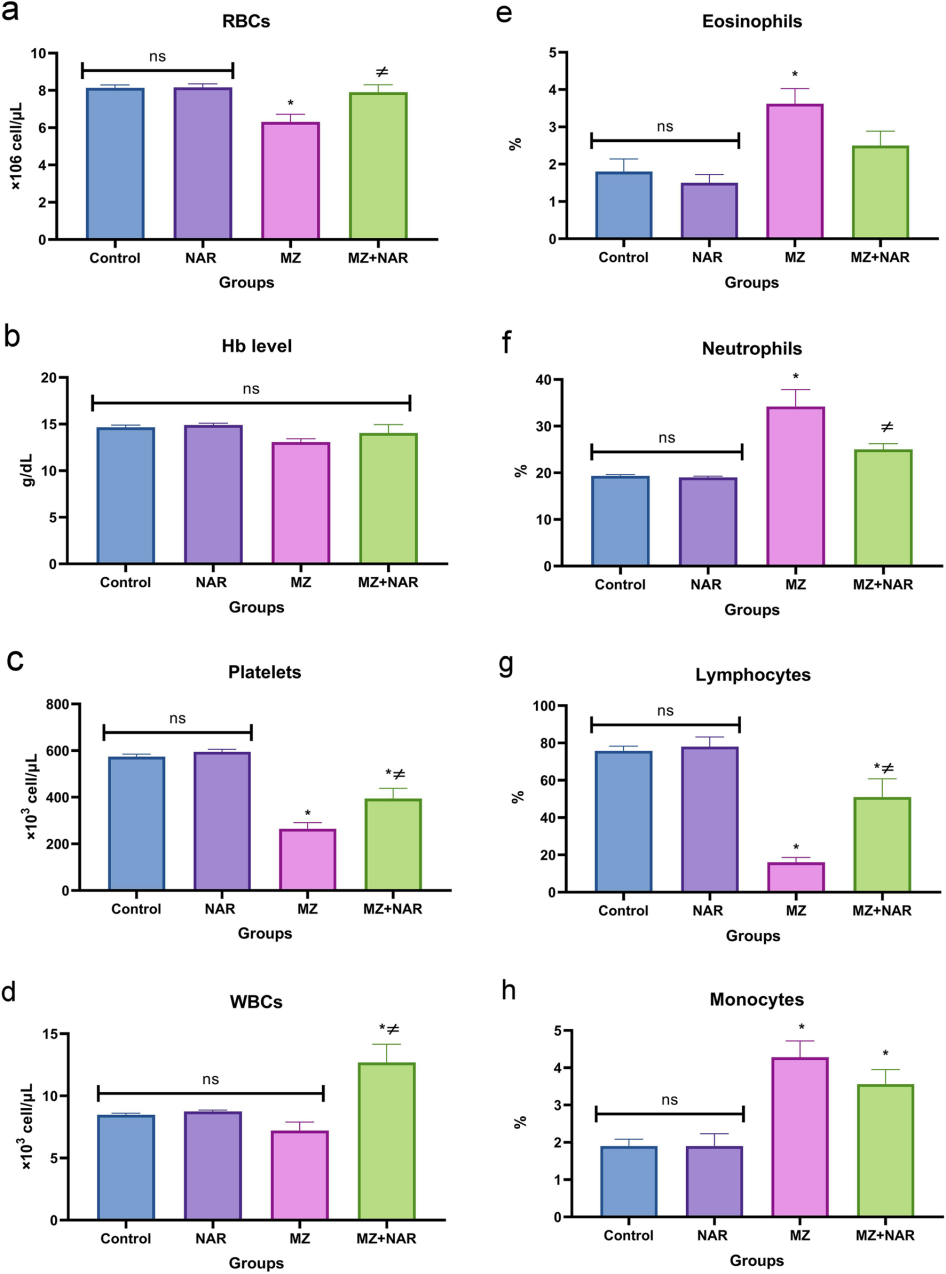
Bar chart represents the effect of MZ and/or NAR on some hematological parameters. **a** RBCs count, **b** Hb level, **c** platelets count, **d** WBCs count, **e** eosinophils, **f** neutrophils, **g** lymphocytes, and **h** monocytes. Values presented as mean ± SEM (n = 7). ⁎ means a significant difference compared to the corresponding control group, while ≠ means a significant difference compared to the MZ group at *P* ≤ *0.05.* *Hb* hemoglobin, *RBCs* red blood corpuscles, *WBCs* white blood corpuscles

Figure 2 presented a normal hematological profile in both the control and NAR groups with no significant difference. While the MZ group reported a significant decrease in RBCs, platelets, and lymphocytes along with an increase in eosinophils, neutrophils, and monocytes in comparison with the control group. On the other hand, the administration of NAR with MZ significantly increased the RBCs, platelets, WBCs, and lymphocytes count and decreased the neutrophils count if compared with the MZ group, but monocytes and eosinophils percentage didn’t show any significant difference in the MZ + NAR group compared with the MZ group.

### Biochemical analysis

**Fig. 3:**
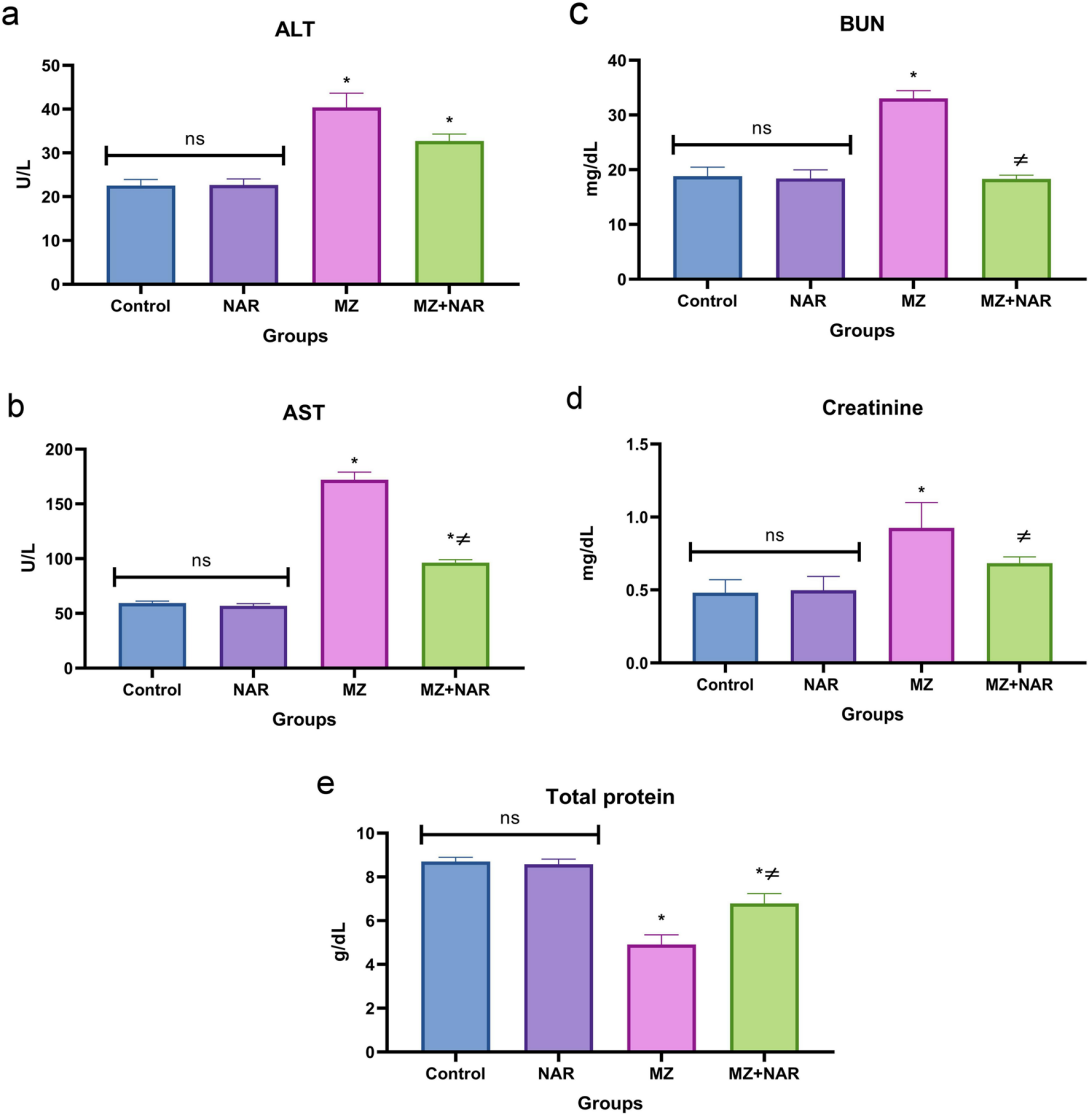
Bar chart represents the effect of MZ and/or NAR on liver and kidney biomarkers level. **a** ALT, **b** AST, **c** BUN, **d** Creatinine, and **e** total proteins. Values are presented as mean ± SE (n = 7). ⁎ means a significant difference compared to the corresponding control group, while ≠ means a significant difference compared to MZ group at *P* ≤ *0.05*

The data presented in Fig. 3 didn’t show any significant difference in ALT, AST, BUN, creatinine, and total protein levels between the control and NAR groups. Otherwise, the MZ group showed a significant increase in the serum levels of liver enzyme activities, BUN, and creatinine alongside decline in total protein levels compared to the control group. Whereas the administration of NAR with MZ significantly reduced the serum activity/level of AST, BUN, and creatinine as well as it elevated the total protein levels compared to the MZ group.

### Oxidative stress assay

**Fig. 4:**
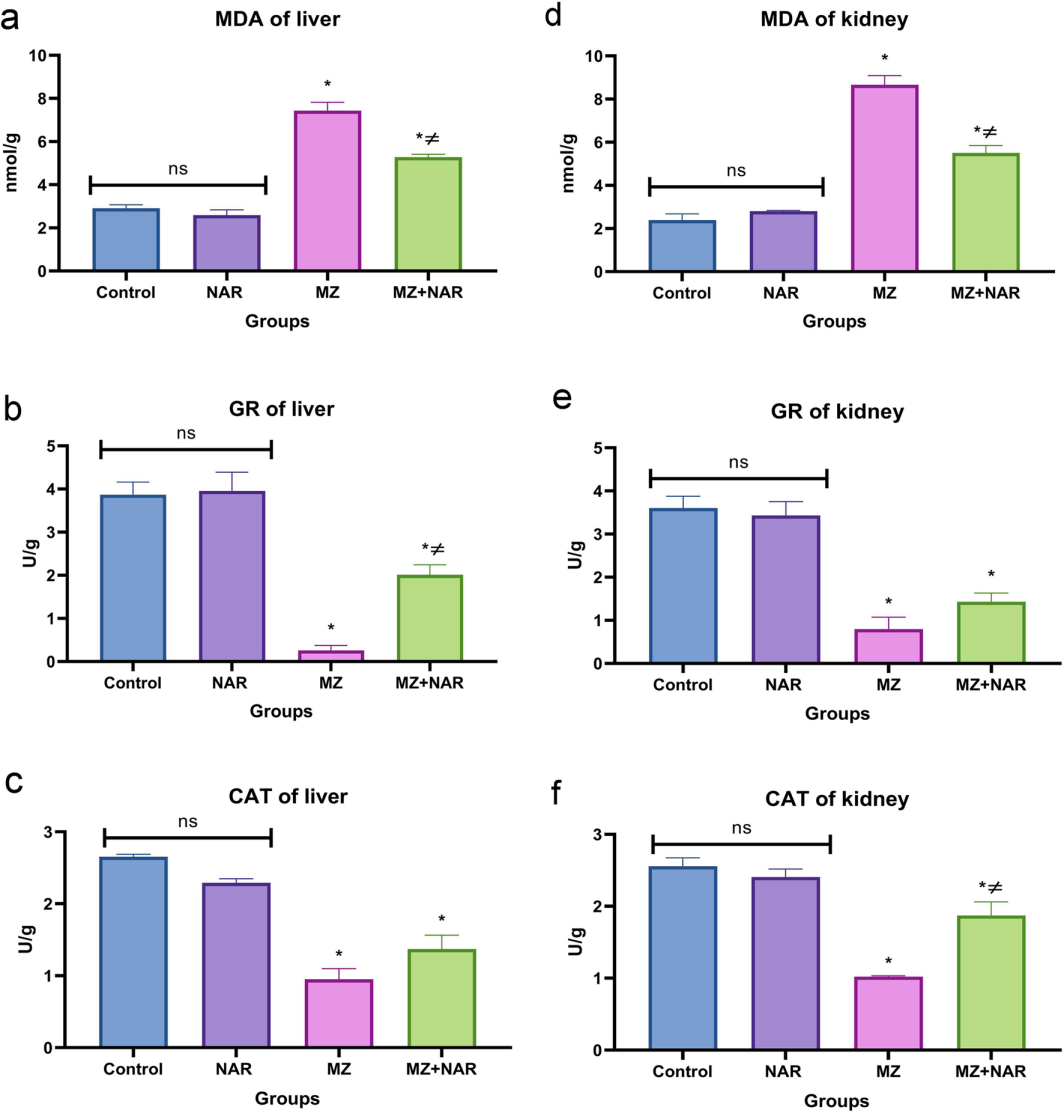
Bar chart represents the effect of MZ and/or NAR on MDA (**a**, **d**), GR activity (**b**, **e**), and CAT activity (**c**, **f**) in both liver and kidney tissues. Values are presented as mean ± SE (n = 7). ⁎ means a significant difference compared to the corresponding control group, while ≠ means a significant difference compared to MZ group at *P* ≤ *0.05*

Figure 4 showed a non-significant difference in both oxidant and antioxidant biomarkers (MDA, GR, and Catalase) between the control and NAR groups. Otherwise, the MZ group noticed a significant elevation in MDA alongside reduction in both GR and Catalase in contrast to the control group. While the MZ + NAR group displayed a marked reduction in MDA and elevation in both GR and Catalase compared with the MZ group.

### Histopathological examination

**Fig. 5:**
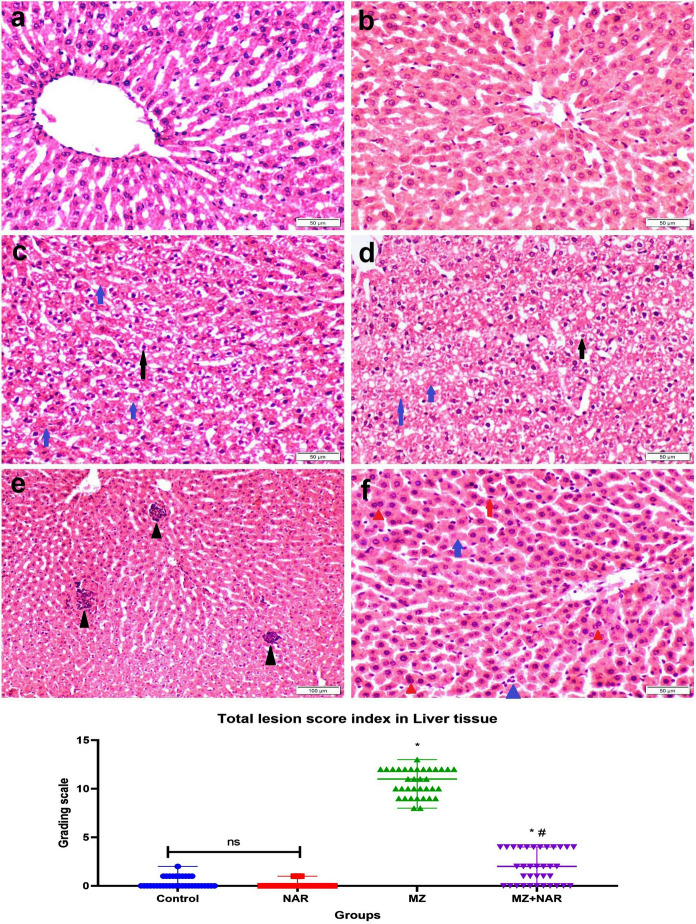
**a**–**f** Photomicrograph of liver sections stained with H&E obtained from various experimental groups. **a** Control negative group, and **b** NAR group showed normal histoarchitecture. **c–e** MZ-exposed groups showed hepatocellular vacuolization (black arrow), necrosis (blue arrow), multifocal inflammatory cells infiltration (black triangle). **f** MZ + NAR group showed sporadic cell necrosis (blue arrow), sinusoidal lymphocytic infiltrations (blue triangle), hyperactivation of Kupffer cells (red arrow), binucleated hepatocytes (red triangle). **g** Scatter plot represents total hepatic lesion score index. Values are presented as median with range (n = 35 images/group). ⁎ means a significant difference compared to the corresponding control group, while ≠ means a significant difference compared to MZ group at *P* ≤ *0.05*. (Color Figure online)

The liver tissue from both the control and NAR groups revealed well-preserved liver architecture, characterized by normal hepatic cords, intact central veins, and normal portal areas. In contrast, liver sections from the MZ-exposed group showed marked pathological changes, including vacuolar degeneration, microvesicular steatosis, hyperactivation of Kupffer cells, and multifocal aggregation of inflammatory cells. Additionally, lymphocytic infiltration was seen within the hepatic sinusoids, showing active hepatic inflammation. Otherwise, the hepatic tissue from the MZ + NAR group showed partial restoration of liver architecture, while hyperactivity of Kupffer cells and sinusoidal lymphocytic infiltration were markedly reduced compared to the MZ group, suggesting a protective and ameliorative effect of the NAR (Fig. 5).

**Fig. 6:**
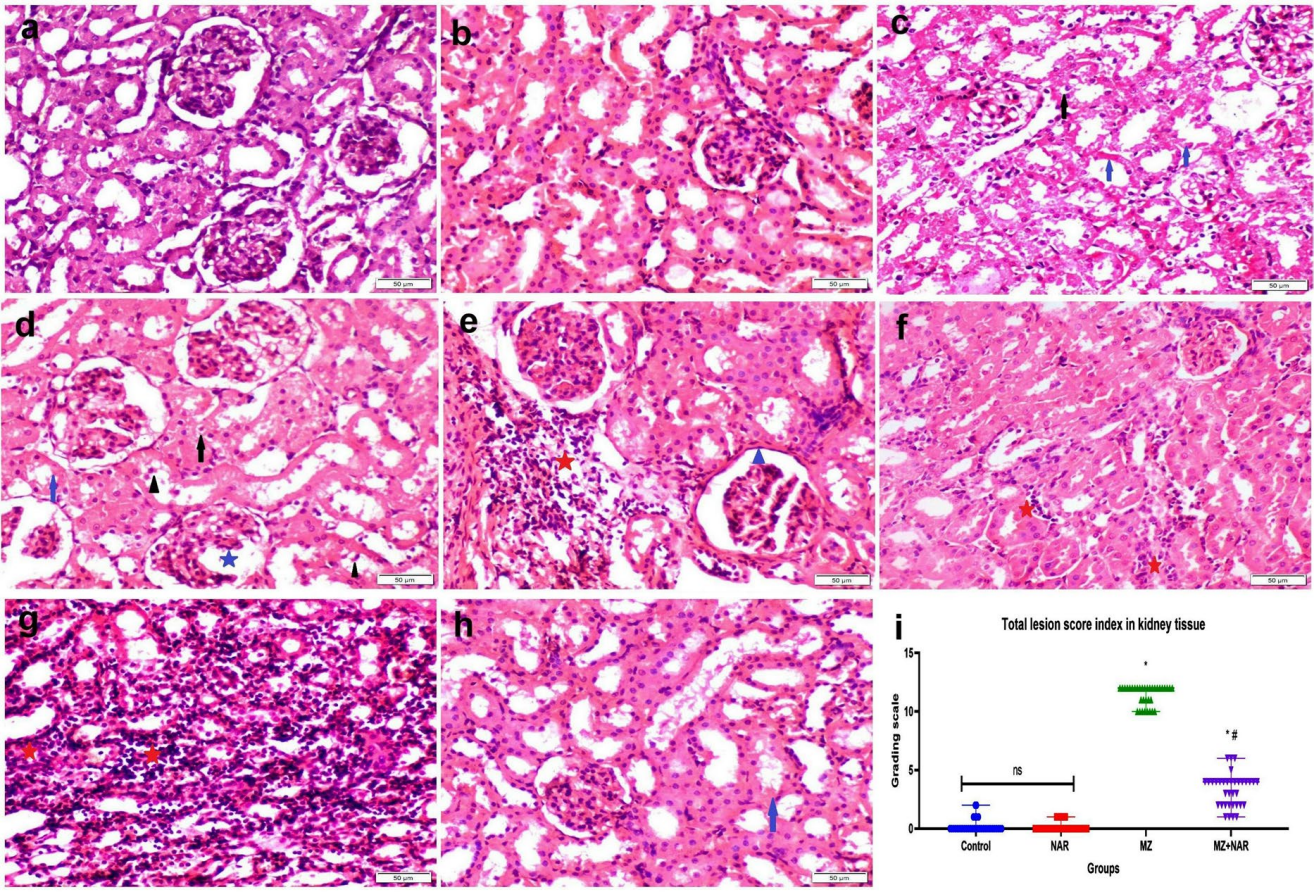
**a**–**h** Photomicrograph of kidney sections stained with H&E obtained from various experimental groups. **a** Control negative group, and **b** NAR group showed normal histoarchitecture. **c**–**g** MZ-exposed groups showed tubular epithelial vacuolization (black arrow) and necrosis (blue arrow), cellular desquamation (black triangle), widening of Bowman’s space (blue star), thickening of Bowman’s capsule (blue triangle), interstitial inflammatory cells infiltration (red triangle). **h** MZ + NAR group showed sporadic cell necrosis of some renal tubular epithelium (blue arrow). **i** Scatter plot represents total renal lesion score index. Values are presented as median with range (n = 35 images/group). ⁎ means a significant difference compared to the corresponding control group, while ≠ means a significant difference compared to MZ group at *P* ≤ *0.05*. (Color Figure online)

The renal tissue sections from both the control and NAR groups showed a preserved healthy histological structure of normal glomeruli, intact renal tubules, and no signs of any degenerative or inflammatory changes. Contrariwise, the renal tissue of the MZ group exhibited extensive glomerular abnormalities, including thickening of the glomerular capsule, significant widening of Bowman’s space, and atrophy of the glomerular tuft. Tubular structures showed several degenerative changes to complete necrosis and desquamation of their epithelial lining alongside luminal cellular debris. The interstitial tissue was heavily infiltrated by inflammatory cells, predominantly macrophages and lymphocytes. In the MZ + NAR group, renal sections showed a degree of structural preservation. Glomeruli appeared normal and tubular degeneration was moderate. Neither inflammatory cells infiltration nor epithelial cell necrosis were seen in any sections (Fig. 6). The results of histopathological lesion scoring showed the highest lesion score index in both liver and kidney of the MZ group. While the lesion score index was markedly reduced in the MZ + NAR group compared to the MZ group, but still higher than the corresponding control group.

### Immunohistochemistry

**Fig. 7:**
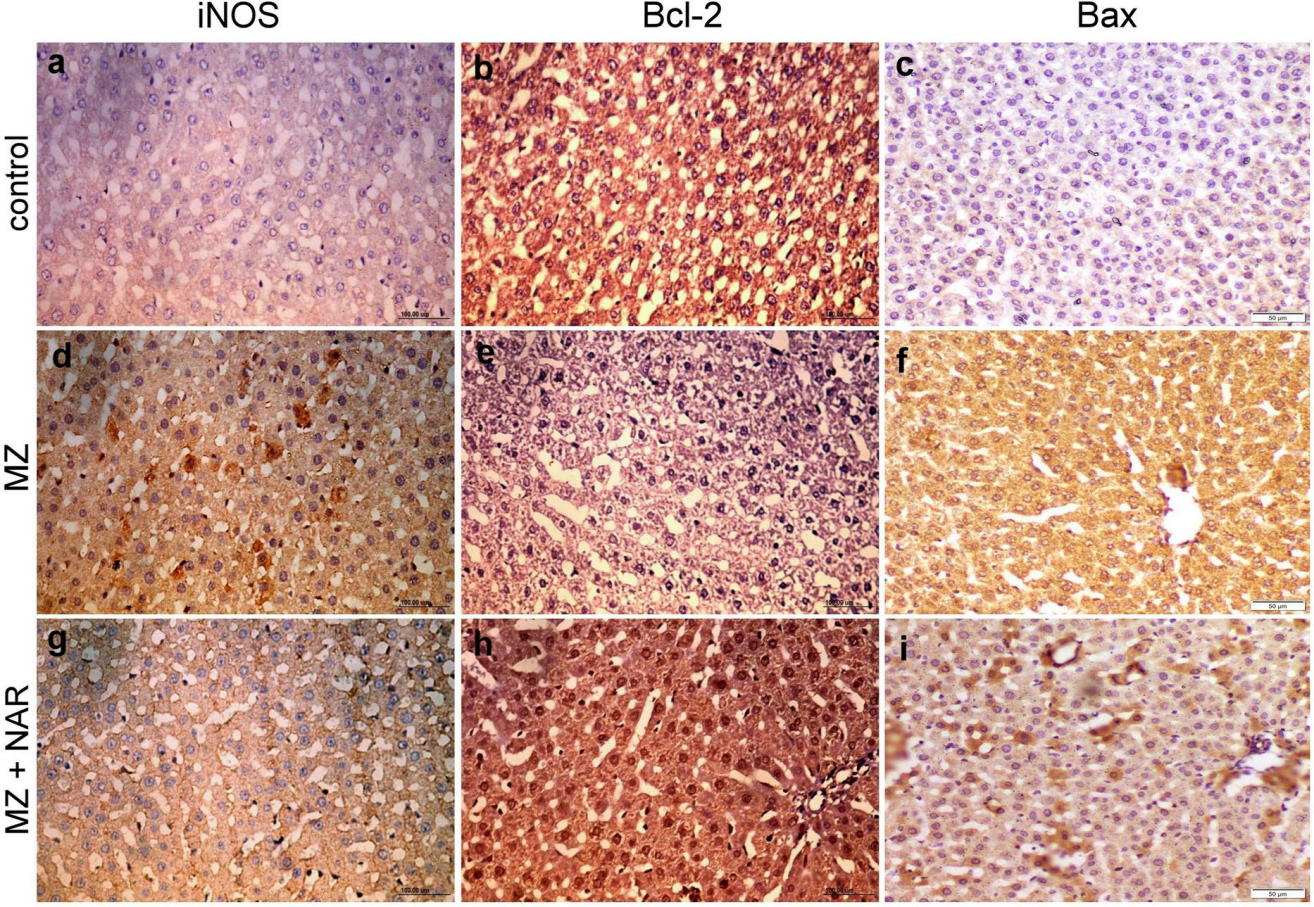
Photomicrograph for the localization of iNOS, Bcl-2, and Bax in liver sections of various experimental groups. **a**–**c** Control group showed negative iNOS and Bax immunoexpression with strong Bcl-2 cytoplasmic localization. **d–f** MZ-exposed groups displayed strong iNOS and Bax immunopositivity with weak Bcl-2 immunoexpression. **g**–**i** MZ + NAR group showed mild to moderate iNOS and Bax immunoexpression alongside strong Bcl-2 cytoplasmic and nuclear localization

**Fig. 8:**
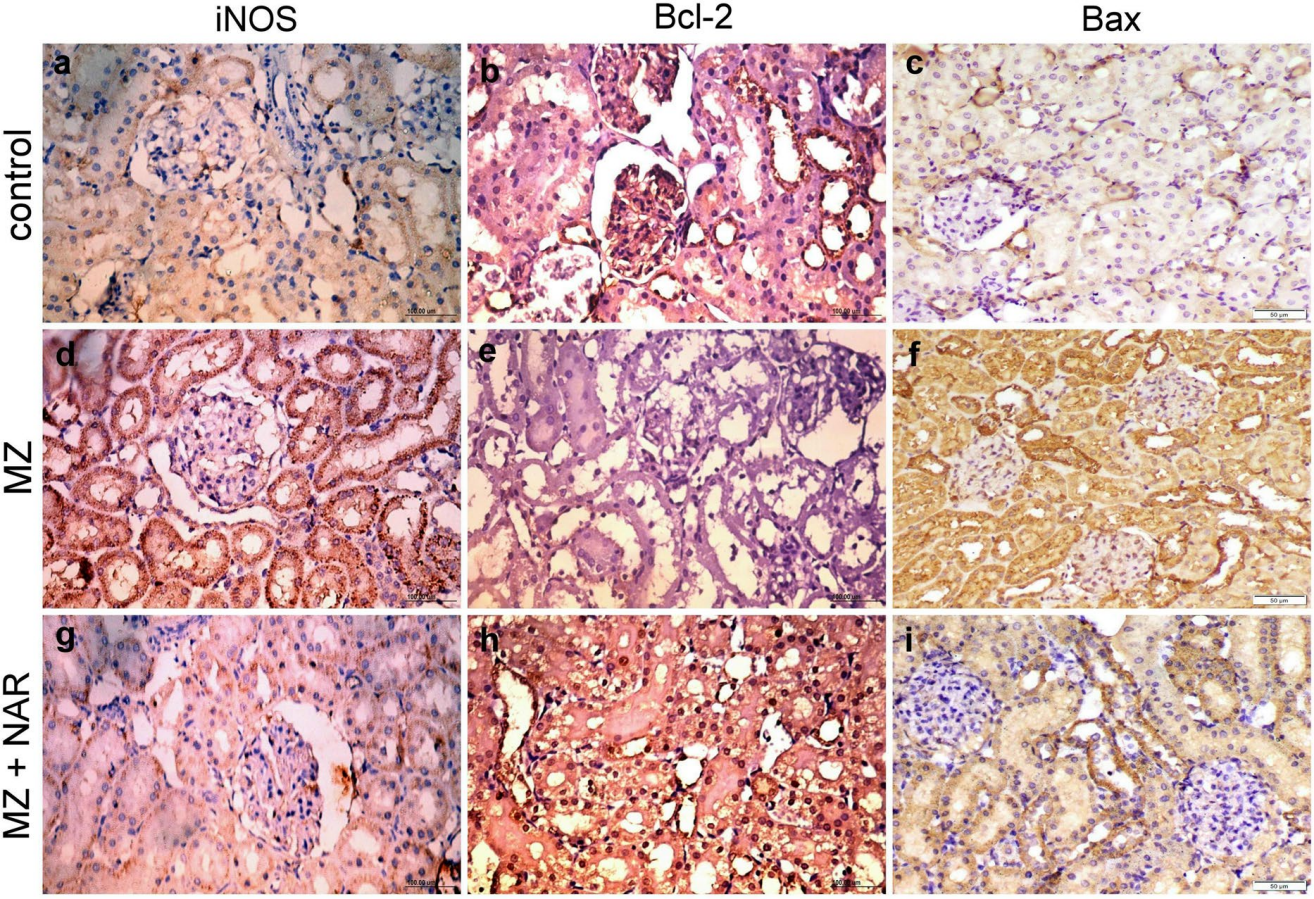
Photomicrograph for the localization of iNOS, Bcl-2, and Bax in kidney sections of various experimental groups. **a**–**c** Control group showed normal baseline localization of iNOS and Bax immune markers with moderate Bcl-2 cytoplasmic localization. **d**–**f** MZ-exposed groups displayed strong iNOS and Bax immunopositivity with negative Bcl-2 immunoexpression. **g**–**i** MZ + NAR group showed mild iNOS and Bax immunoexpression alongside strong Bcl-2 cytoplasmic and nuclear localization

**Fig. 9:**
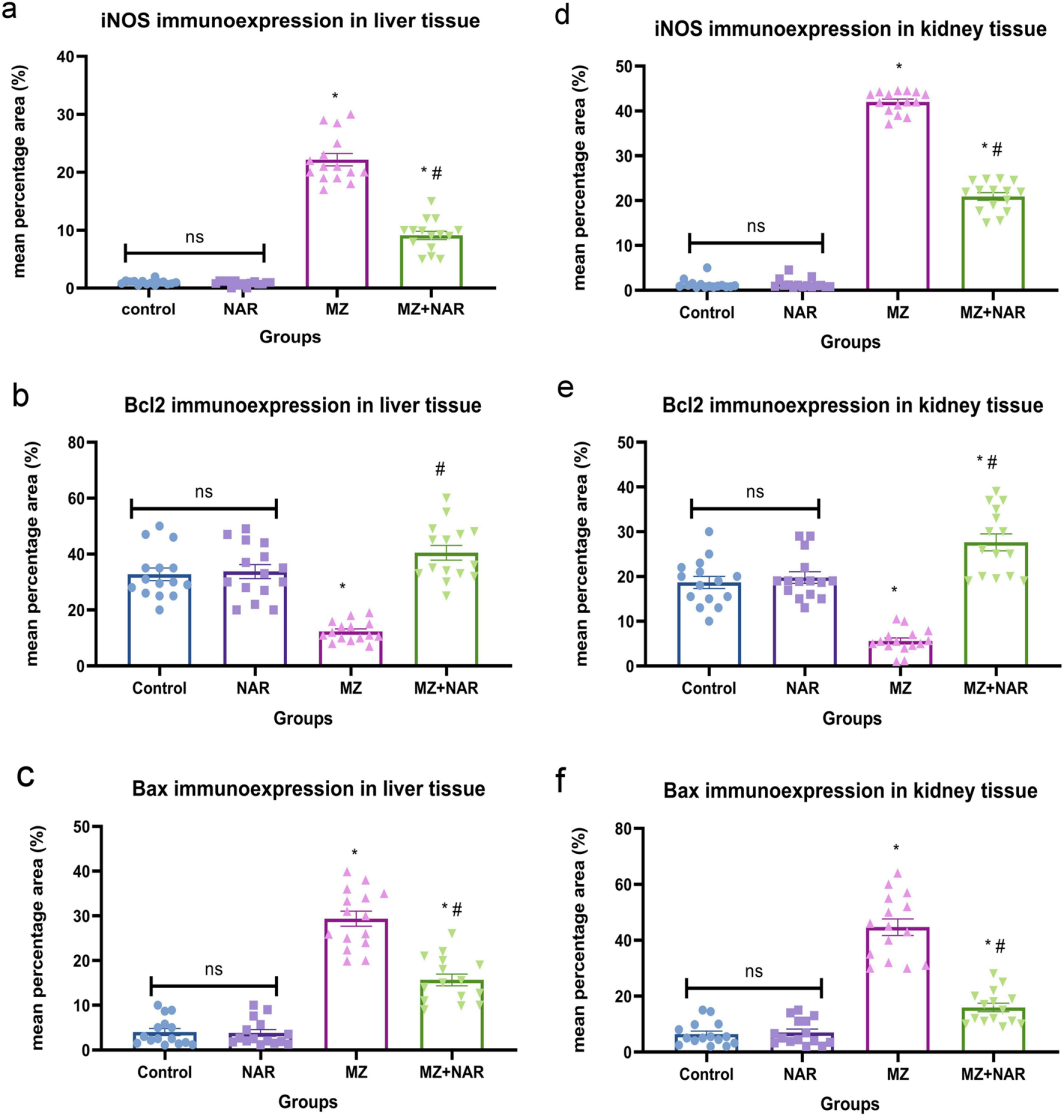
Scatter plot bar represents the mean percentage positive area for iNOS (**a**, **d**), Bcl2 (**b**, **e**), and Bax expressions (**c**, **f**). Values are presented as mean ± SE (n = 15 images/group). ⁎ means a significant difference compared to the corresponding control group, while ≠ means a significant difference compared to MZ group at *P* ≤ *0.05*

Immunohistochemical analysis of the hepatorenal tissue in the control and NAR-treated groups showed normal expression patterns of iNOS, Bax, and BCL-2. Otherwise, the MZ-exposed group showed strong immunopositivity for iNOS and Bax alongside marked reduction in BCL-2 expression. However, a marked reduction in iNOS and Bax expression and elevation of BCL-2 immunoexpression was seen in the MZ + NAR group compared to the MZ group (Figs. 7 and 8). Quantitative immunohistochemical scoring revealed that the MZ group exhibited the highest mean positive area for iNOS and Bax, with a concurrent decrease in BCL-2 expression, in both liver and kidney. Compared to the MZ group, the MZ + NAR group showed a marked reduction in the mean positive area for iNOS and Bax, along with a significant elevation in BCL-2 expression (Fig. 9).

### Elemental analysis

**Fig. 10:**
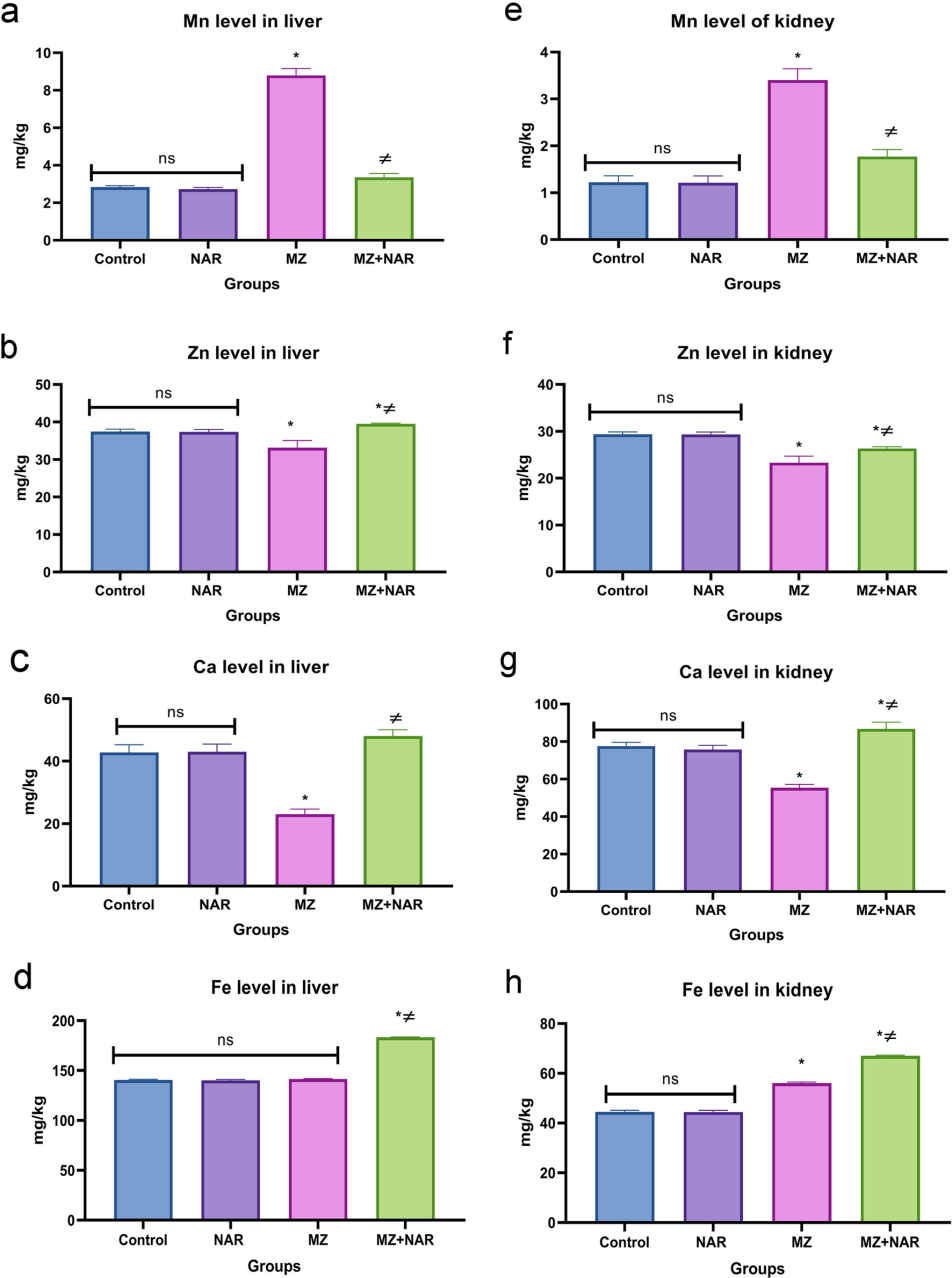
Bar chart represents the effect of MZ and/or NAR on Mn (**a**, **e**), Ca (**b**, **f**), Zn (**c**, **g**), and Fe (**d**, **h**) levels in hepatorenal tissues. Values are presented as mean ± SE (n = 7). ⁎ means a significant difference compared to the corresponding control group, while ≠ means a significant difference compared to MZ group at *P* ≤ *0.05*

All elements including Mn, Zn, Fe, and Ca levels were not significantly different in both the control and NAR groups. While MZ administration significantly increased Mn but decreased Ca and Zn levels in the liver and kidneys compared to the control group. Liver Fe content in the MZ group was not significantly different from the control group, but kidney Fe content was significantly elevated. Co-administration of NAR with MZ significantly increased Ca and Zn levels in both liver and kidneys compared with either MZ or NAR alone. The MZ + NAR group also had a higher liver Fe content than either MZ or NAR alone, while kidney Fe level was significantly elevated versus the MZ group but reduced versus the NAR group. Mn levels in both liver and kidney significantly decreased in the MZ + NAR group compared with the MZ group, showing no significant difference compared to the NAR group (Fig. 10).

### Quantitative RT-PCR analysis of* MT-1*,* CYP1A1*, and* caspase-3* expression

**Fig. 11:**
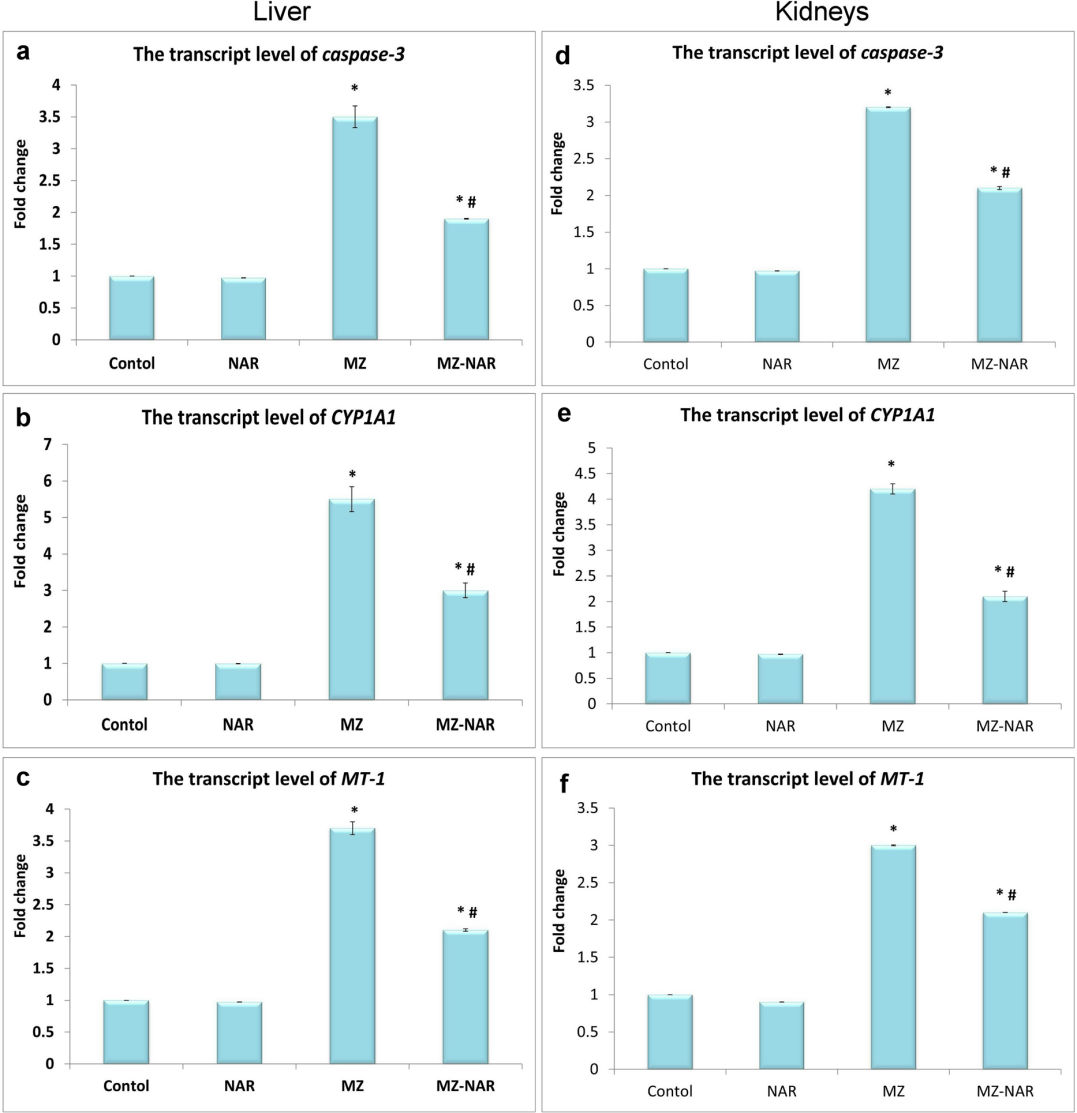
Bar chart represents the effect of MZ and/or NAR on the transcriptase levels of caspase-3 (**a**, **d**), CYP1A1 (**b**, **e**), and MT-1 (**c**, **f**) of both liver and kidney tissues. All data are presented as mean ± SE (n = 7). ⁎ means a significant difference compared to the corresponding control group, while ≠ means a significant difference compared to the MZ group at *P* ≤ *0.05*

The MZ administration significantly elevated the transcriptase levels of *caspase-3, MT-1,* and *CYP1A1* genes in both liver and kidney tissues. Otherwise, co-administration of NAR with MZ significantly improved the toxic of MZ by downregulating the hepatorenal mRNA expression levels of *caspase-3, MT-1,* and *CYP1A1* genes (Fig. 11).

## Discussion

Overexposure to pesticides results in harmful environmental consequences which are reflected on humans, animals, soil, and crops (Zhou et al. 2024). Although MZ is widely used as a fungicide in agriculture and another different fields and many countries do not yet classify it as toxic pesticide, its toxicity has been proved in several experimental studies that reveal its harmful properties which require careful attention (Dall'Agnol et al. 2021). So, the present study aimed to investigate the toxic effect of repeated MZ dosing (1/20 LD50) on the liver and kidney of rats focusing on the role of some biometals such as Mn, Zn, Fe, and Ca, besides to evaluate the significant anti-toxic effects of NAR against such toxicity. In the present study, the dose of MZ was selected based on LD50 instead of food residual levels for three reasons. First, the residual levels of MZ in food are inconsistent; one study found high levels (8.1–17.72 mg/kg) in papaya (Ahuja and Mohapatra 2010), while others reported a wide range levels (0.93–4.13 mg/kg) in corn (Mandal et al. 2022). Second, lack of detailed reports from some countries complicates dosage selection in toxicology studies due to potential for higher human exposure. Third, sublethal doses of MZ are commonly used in many subacute toxicological studies, while the food residual levels of MZ are more applicable in chronic study to investigate long-term effects (Shekhar et al. 2024). LD₅₀, the standard measure for acute toxicity, was used to determine sublethal experimental doses, typically 1/10–1/100 of the LD₅₀ (EFSA et al. 2020; Kumawat et al. 2024). MZ has high oral LD₅₀ (5000 mg/kg) in rats, indicating low acute lethality (Yahia et al. 2019).

Our findings revealed a significant deterioration in the hematological profile of MZ group suggesting its hematotoxic effect. We agreed with a recent study by Hashem et al. (2018), who confirmed eosinophilia and lowering RBCs count in rats after administering 300 mg/kg MZ for 2 weeks. MZ-mediated low RBCs count, and thrombocytopenia could be explained by the shortening of the lifespan of circulating RBCs and increasing the rate of RBC destruction (Quds et al. 2023). Thrombocytopenia may also be attributed to a combination of several factors, including immune-mediated toxicity, oxidative stress damage, and bone marrow suppression (Kumawat 2020). Furthermore, MZ-induced lymphocytopenia may be the result of diminished lymphocyte proliferation, increased immune cell apoptosis, or impaired function of lymphoid organs (Mandarapu and Prakhya 2016). Additionally, the observed neutrophilia and monocytosis in MZ group may reflect the oxidative stress-mediated inflammatory response that was induced by MZ (Kumboyono et al. 2024). Otherwise, the coadministration of NAR with MZ greatly improved the hematological profile of rats. Several recent studies demonstrated that the NAR treatment significantly improved the hematological parameters such as RBCs count, platelet levels, total and differential leukocytic counts via reducing oxidative stress markers and enhancing antioxidant enzyme activities (Hambardikar and Mandlik 2022; Temel 2023). In our study, lymphocytosis and elevated WBCs count may be explained by the fact that NAR could influence the immune functions and support hematological health under toxic conditions (Tang et al. 2024; Huang et al. 2024).

In this study, MZ-induced hepatic and renal damage was evidenced by elevated liver enzyme activity and kidney function biomarker levels. Increased serum ALT and AST activity indicated hepatocellular leakage and compromised cell membrane integrity, while elevated BUN and creatinine levels marked significant nephron damage (Aprioku et al. 2023; Thakur et al. 2024). These findings were matched with the histopathological results that proved extensive cellular degeneration and necrosis in both the hepatic and renal tissues of the MZ group. Our findings agreed with several studies which confirmed that MZ caused an elevation in serum liver enzyme activities, suggesting hepatocellular or cholestatic damage (Chiali et al. 2020; Gök and Deveci 2022; Nuchniyom et al. 2023). Several studies have reported significant elevations in serum creatinine, BUN levels along with tubular cell necrosis, vacuolization, vascular congestion, and infiltration of inflammatory cells in renal tissues following MZ exposure, suggesting renal dysfunction (Kumawat 2020; Mesallam et al. 2023). These alterations can be mechanistically linked to the oxidative stress-mediated degradation of membrane lipids (Mohideen et al. 2023). As increased MDA levels reflect enhanced lipid peroxidation of cellular and organelle membranes, a process where ROS degrade polyunsaturated fatty acids in cell membranes (Endale et al. 2023). Moreover, it contributes to cellular toxicity through several mechanisms, including protein and DNA modification, mitochondrial dysfunction, activation of inflammatory pathways, and disruption of redox homeostasis (Mohideen et al. 2021; Cordiano et al. 2023). In addition, the depletion of antioxidant enzymes activity (GR and catalase) suggests that MZ overwhelmed the hepatorenal antioxidant defense leading to increased oxidative stress and potential cellular injury (Liu et al. 2022; Quds et al. 2023). In agreement with our findings, many recent studies found that exposure to MZ led to increased renal and hepatic MDA content and decreased levels of GSH and catalase, which showed impairments of hepatorenal function by disrupting oxidative phosphorylation pathways (Zhang et al. 2023; Yousuf et al. 2024). Another aquatic study confirmed that exposure to MZ led to significant increases in ROS and MDA levels, along with decreased activities of antioxidant enzymes (Khan et al. 2025). On the other hand, co-administration of NAR with MZ normalized liver/kidney biomarkers, improved microscopic appearance, lowered MDA, and increased GR/catalase activity versus MZ alone. This suggests reduced oxidative stress damage and boosted antioxidant defenses. These effects are likely mediated by naringin’s potent free radical scavenging capacity and its regulatory influence on redox homeostasis within hepatorenal tissues (Gelen et al. 2022). Several studies confirmed that NAR mitigates oxidative stress damage by enhancing the antioxidant defense system reduced oxidative stress markers (Hassan et al. 2021; Xi et al. 2023; Fayaz et al. 2025).

Furthermore, our immunohistochemical results also revealed that oxidative stress has a vital role in MZ-induced hepatorenal damage. The positive iNOS immunoreactivity that was noticed in the MZ group confirmed the upregulation of oxido-inflammatory stress pathways (Liu et al. 2023). iNOS is typically induced during inflammatory conditions and contributes to the overproduction of nitric oxide (NO), which can react with superoxide radicals to form peroxynitrite, a potent oxidant that causes lipid peroxidation, protein nitration, and DNA damage, that induce cellular injury and apoptosis (Quan et al. 2017). We also found increased expression of the pro-apoptotic marker (Bax, Caspase-3) and decreased the anti-apoptotic marker (Bcl-2) in the MZ group, suggesting mitochondrial-dependent apoptosis (Li et al. 2024). One study revealed that MZ exposure led to a significant cellular damage induced by upregulated iNOS expression and increased oxidative stress markers (Mohammadi-Sardoo et al. 2018). Another study revealed that MZ treatment resulted in activation of Bax/Bcl-2 apoptotic pathways indicating a shift towards apoptosis via the mitochondrial pathway (Lori et al. 2021). While co-treatment of NAR with MZ displayed week iNOS and Bax expression with strong Bcl-2 immunoexpression in both liver and kidney sections. The downregulation of iNOS reflects the antioxidant and anti-inflammatory capacity of NAR thereby protecting cellular structures from lipid peroxidation, protein nitration, and DNA fragmentation (Shi et al. 2023; Caligiuri et al. 2023). While the shift in the apoptotic balance suggests that NAR mitigated apoptosis by restoring redox homeostasis and preventing oxidative damage (Cui et al. 2018). NAR effectively reduces oxidative stress and apoptosis in tissue injury models by downregulating iNOS expression and modulating the Bax/Bcl-2 ratio (Guo et al. 2017; Zhao et al. 2022; Mohamed et al. 2023).

Additionally, our study proposed that MZ, as a manganese-containing fungicide, contributes to the accumulation of Mn in hepatorenal tissue. Whereas Mn accumulation may disturb the homeostasis of other essential trace elements, including Zn, Ca, and Fe. We suggest that elemental imbalance is likely to play a critical role in the pathogenesis of MZ-induced hepatorenal toxicity. The excessive aggregation of Mn in both liver and kidney tissues induced further oxidative stress damage via ROS overgeneration and impairment of antioxidant enzymatic activities leading to cellular injury (Pradhan et al. 2023; Mattison et al. 2024). Additionally, Mn disrupts mitochondrial function and impairs cellular energy production (Dorman 2023). It may also interfere with the homeostasis of essential trace elements such as Ca, Fe, and Zn, further contributing to metabolic imbalance, cellular dysfunction resulted in tissue damage (Chen et al. 2025). Moreover, reduced Ca may affect cellular signaling and membrane stability, while altered Fe and Zn levels can compromise immune function and antioxidant enzyme activity, exacerbating MZ-induced cellular stress and toxicity (Gensluckner et al. 2024). A recent study proved that low-dose MZ exposure in rats led to a significant increase in Mn level in the liver and renal cortex, which was associated with oxidative damage and tubular injury (Akhtar and Trombetta 2023; Liang et al. 2024). The mechanism underlying the role of biometals in MZ-induced hepatorenal toxicity confirmed by the upregulation of *MT-1 and CYP1A1* gene levels in both liver and kidney tissues of the MZ group. MT-1 gene is a key regulator of trace element homeostasis and a strong indicator for metal toxicity (Nordberg and Nordberg 2025). Moreover, MT-1 is involved in binding and detoxifying harmful intracellular metal ions to reduce oxidative stress damage (Chiaverini and De Ley 2010). So, elevated MT-1 expression appears to be a cellular effort to counteract either MZ or Mn-induced oxidative stress damage. CYP1A1 is a member of cytochrome P450 enzymes that metabolize MZ and considered as an indicator for cellular integrity (Stading et al. 2020). The overactivation of CYP1A1 may be attributed to oxidative stress injury.

Moreover, the present study explored the chelating effect of NAR against Mn bio-aggregation in hepatorenal tissue and its ability to restore other elemental balance. The observed normalization of mineral levels in the NAR + MZ group shows that NAR may exert a regulatory influence on metal homeostasis, potentially through antioxidant activity and modulation of metal transport mechanisms (Jahanshahi et al. 2021, 2022). The elevated Ca levels may help to support membrane integrity and intracellular signaling, both of which are typically compromised during oxidative and metal-induced stress (Ning et al. 2021). While the NAR + MZ group had higher Ca content than the control group, particularly in kidney tissues, it remained within the normal range (liver: 45 ± 2.4 mg/kg; kidney: 75 ± 5.7mg/kg), as reported in a recent study (Orct et al. 2017). The elevation of Ca levels is likely due to interaction of MZ with NAR, rather than either MZ or NAR itself, as evidenced by the lack of change in the NAR control group or MZ group. But this increase had no adverse effects on hepatorenal function, as evidenced by stable liver enzyme activity, kidney biomarker levels, and microscopic examination of the organs. Moreover, the reduction in the hepatic and renal content of Mn in the MZ + NAR group may be contributed to the maintenance of mitochondrial and enzymatic activity as well as reduce the oxidative stress damage of liver and kidneys (Mezzaroba et al. 2019). Earlier studies have showed that the antioxidant flavonoids—of which NAR is one—can influence metal bioavailability, either by direct chelation or through modulation of metal transporters, thus protecting tissues from heavy metal-induced toxicity (Jahanshahi et al. 2022; Ferdigg et al. 2025). From molecular insight, the MZ + NAR group showed a significant downregulation of *MT-1, CYP1A1*, and *casp-3* gene levels, confirming the protective role of NAR on MZ-induced hepatorenal toxicity. The downregulation of MT-1 expression demonstrated that oxidative stress was substantially alleviated, reducing the requirement for stress-responsive defense, and enhancement of metal toxicity (Yang et al. 2024). While the downregulation of CYP1A1 indicates a reduced need for excessive detoxification activity, likely due to ability of NAR to neutralize free radicals and intracellular metal ions, which verified improvement of cellular integrity (Santes-Palacios et al. 2016). Likewise, lower caspase-3 level suggests that NAR mitigated apoptosis and preserving cellular integrity too (Khalil et al. 2012).

The present study had some limitations, which can be resolved in further studies. The study used sublethal dose of MZ (1/20 LD50) to determine the subacute toxicity of MZ on liver and kidneys. The question of whether findings from high-dose animal studies are applicable to significantly lower human exposure levels is an important challenge in toxicology. So, further studies required to determine the short- and long-term effect of residue-based dosing on various organs at different time points. Additionally, further studies are needed to assess the effect of MZ-NAR interaction on Ca levels in serum and various organs, and to investigate the potential contributions of NAR, MZ, or both to dystrophic calcification. Comparative studies in multiple animal models are needed to validate the role of biometals in MZ-toxicity, complemented by human cell culture models to better translate findings from rats to humans.

## Conclusion

The results of the current study concluded that the daily exposure of rats to sublethal dose of MZ induced hematological disturbances, oxidative stress-mediated functional and structural damage in liver and kidneys. MZ can induce oxidative stress either directly via ROS overgeneration and exhaustion of antioxidant enzymatic activity or indirectly via interfering with some metal homeostasis such as Mn, Ca, Zn, and Fe, which confirmed by upregulation of *MT-1* and *CYP1A1* gene levels. However, the co-administration of NAR with MZ significantly reversed these toxic effects via restoring the antioxidant enzyme activity, improving the hematological profile, rebalancing essential element levels, modulating the iNOS expression, restoring Bax/Bcl-2 equilibrium, and downregulating various genes like *MT-1*, *CYP1A1*, and *Caspase-3.* These results support the protective role of NAR against MZ-induced hepatorenal toxicity through the potent antioxidant, anti-inflammatory, anti-apoptotic, and chelating effects of NAR.

## Data Availability

All data will be made available on request.
